# Xanthohumol, a Prenylated Chalcone from Hops, Inhibits the Viability and Stemness of Doxorubicin-Resistant MCF-7/ADR Cells

**DOI:** 10.3390/molecules22010036

**Published:** 2016-12-28

**Authors:** Ming Liu, Hua Yin, Xiaokun Qian, Jianjun Dong, Zhonghua Qian, Jinlai Miao

**Affiliations:** 1 Key Laboratory of Marine Drugs, Ministry of Education, School of Medicine and Pharmacy, Ocean University of China, Qingdao 266003, China; 2 State Key Laboratory of Biological Fermentation Engineering of Beer (In Preparation), Qingdao 266061, China; yinhua@tsingtao.com.cn (H.Y.); 15133634098@163.com (J.D.); qianzh@tsingtao.com.cn (Z.Q.); 3 College of Food Science and Engineering, Jiangxi Agricultural University, Nanchang 330045, China; azrsqxk97@163.com; 4 Key Laboratory of Marine Bioactive Substance, the First Institute of Oceanography, State Oceanic Administration, Qingdao 266061, China

**Keywords:** xanthohumol, doxorubicin resistance, viability, stemness

## Abstract

Xanthohumol is a unique prenylated flavonoid in hops (*Humulus lupulus* L.) and beer. Xanthohumol has been shown to possess a variety of pharmacological activities. There is little research on its effect on doxorubicin-resistant breast cancer cells (MCF-7/ADR) and the cancer stem-like cells exiting in this cell line. In the present study, we investigate the effect of xanthohumol on the viability and stemness of MCF-7/ADR cells. Xanthohumol inhibits viability, induces apoptosis, and arrests the cell cycle of MCF-7/ADR cells in a dose-dependent manner; in addition, xanthohumol sensitizes the inhibition effect of doxorubicin on MCF-7/ADR cells. Interestingly, we also find that xanthohumol can reduce the stemness of MCF-7/ADR cells evidenced by the xanthohumol-induced decrease in the colony formation, the migration, the percentage of side population cells, the sphere formation, and the down-regulation of stemness-related biomarkers. These results demonstrate that xanthohumol is a promising compound targeting the doxorubicin resistant breast cancer cells and regulating their stemness, which, therefore, will be applied as a potential candidate for the development of a doxorubicin-resistant breast cancer agent and combination therapy of breast cancer.

## 1. Introduction

Breast cancer is a common malignant tumor in woman worldwide. Chemotherapy using doxorubicin (DOX) and docetaxel is still one of the clinically effective treatment strategies for breast cancer. However, the efficacy of DOX is limited by its side effects and the drug resistance, which is the major reason for the failure of breast cancer treatment [1]. The cancer stem-like cells are more resistant to standard chemotherapy drugs [2] and contribute significantly to the drug resistance. Breast cancer stem-like cells are a small population of mostly resting cells defined by their long life, high clonogenicity, self-replicating potential, plasticity, and also drug resistance [3]. Novel strategies are needed to overcome the drug resistance and chemical therapies targeting the breast cancer stem-like cells may contribute a novel method [2]. In recent years, plant-derived compounds and their derivatives [4,5] have been reported to down-regulate the cancer stem-like cells and attenuate the drug resistance, and now phytochemicals in foods or traditional medicines have become one of the cancer multi-therapy approach choices [6].

**Figure 1:**
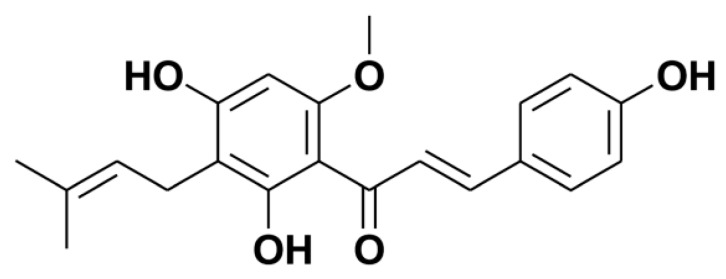
Chemical structure of xanthohumol (XN).

Xanthohumol (XN, Figure 1), a principal prenylated chalcone from the female inflorescences of the hop plant (hops, *Humulus lupulus* L.), is an ingredient of beer [7]. XN has a good safety profile and possesses many beneficial health effects, which has been recently reviewed by Liu and his colleagues [8]. Of note, XN is a potential drug candidate to prevent and treat many kinds of cancers [9,10]. For example, XN is useful for inhibiting the growth of breast cancer MCF-7 cells [10] and inducing apoptosis in MCF-7 cells [11]. The mechanisms of its anticancer activity have been identified, including the inhibition of the initiation and the development of carcinogenesis, the induction of apoptosis, and the inhibition of angiogenesis [9]. Moreover, some results also indicate that XN possibly is a potent chemo- and radio-therapy sensitizer. For example, XN sensitizes DOX resistant MCF-7/ADR cells to the radiation treatment [11]; XN markedly augments the anticancer activity of tumor necrosis factor-related apoptosis-inducing ligand (TRAIL) and sensitizes TRAIL-resistant cancer cells in HeLa [12] and LNCaP cells [13]. XN is also an inhibitor of the efflux transporters, further indicating its potential application in the reverse of multidrug resistance [14]. Nevertheless, the synergic effects in combination with the chemotherapy agents, e.g., DOX, and the possible mechanisms have yet to be further studied.

In this study, we revealed the sensitivity of MCF-7/ADR cells to XN and the potent synergy effect of XN when combined with DOX. Moreover, we tried to illustrate the mechanism was related to the down-regulation of the cancer stem-like characters of MCF-7/ADR cells.

## 2. Results

### 2.1. XN Inhibits Viability, Induces Apoptosis, and Arrests Cell Cycle in MCF-7/ADR Cells

**Figure 2:**
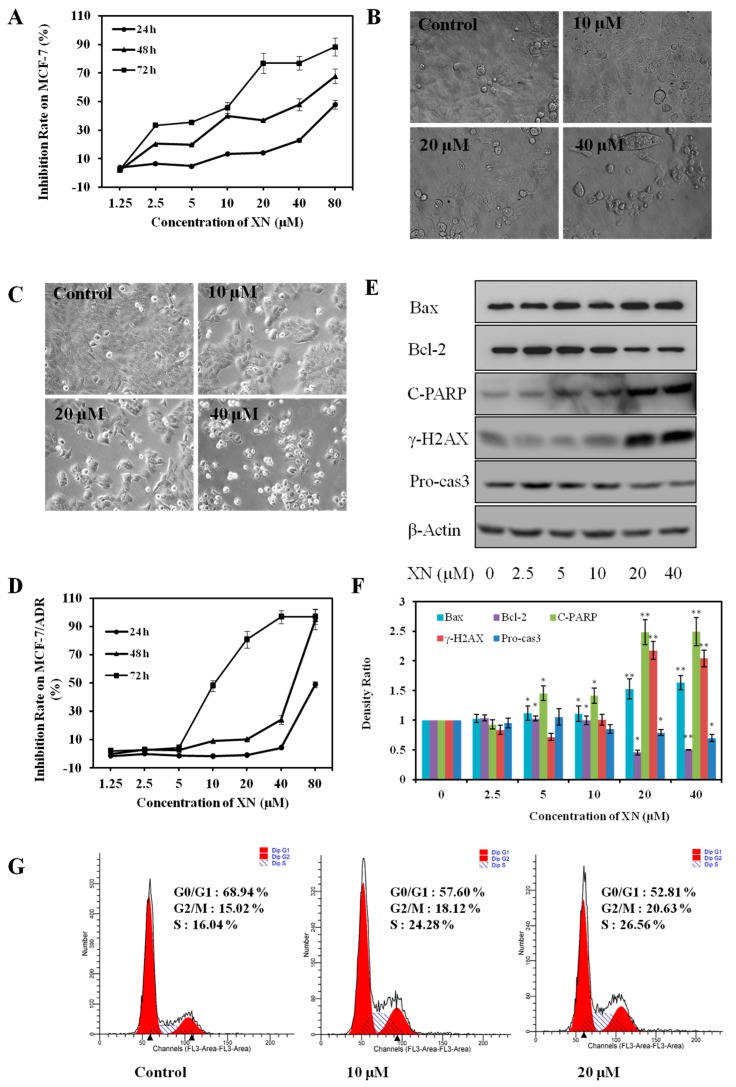
Both MCF-7 and MCF-7/ADR cell lines are sensitive to XN. (**A**) XN inhibits the viability of MCF-7 cells in a concentration- and time-dependent manner. Cells were treated with indicated concentrations of XN for 24, 48, and 72 h, respectively, and then tested by MTT assay; (**B**) XN decreases the population of MCF-7 cells in vitro (×400); (**C**) XN decreases the population of MCF-7/ADR cells in vitro. Cells were treated with XN (0–40 μM) for 48 h in 6-well plate, and observed by inverted microscope (400×); (**D**) XN inhibits the viability of MCF-7/ADR cells in a concentration- and time-dependent manner. Cells were treated with indicated concentrations of XN for 24, 48, and 72 h, respectively, and then tested by MTT assay; (**E**) XN induces apoptosis in MCF-7/ADR cells. Cells were treated with XN (0–40 μM) for 48 h, and the apoptosis related proteins were detected by Western blotting; (**F**) protein levels quantified by scanning densitometry and normalized to control. Results are expressed as the ratio of control. Data represent means ± SD from three independent experiments. * p < 0.05, ** p < 0.01 for control cells versus XN treated cells; and (**G**) XN affects the cell cycle distribution in MCF-7/ADR cells. Cells were treated with XN (0–20 μM) for 24 h, fixed, stained with PI, and detected by flow cytometry.

To evaluate the sensitivity of MCF-7 and MCF-7/ADR cell line to XN, we first examined the growth inhibition effect. In consistent with the previous work [10], our present data also showed XN decreased the cell population and inhibited the viability of MCF-7 cells both in a concentration- and time-dependent manner (Figure 2A,B), with the IC_50_ values of 81.45 ± 6.91, 34.02 ± 3.45, and 11.22 ± 0.95 μM after treatment for 24, 48, and 72 h, respectively. Similarly, as shown in Figure 2C, morphological observation revealed that treatment of MCF-7/ADR cells with XN resulted in markedly decreased cell population and obvious cell shrinkage. The viability of MCF-7/ADR cells was inhibited both in a concentration and time dependent-manner (Figure 2D), and the IC_50_ value of XN against MCF-7/ADR cell lines was 78.33 ± 7.30, 33.71 ± 3.12, and 11.37 ± 1.15 μM with the treatment of XN for 24, 48, and 72 h, respectively. These data revealed that both MCF-7/ADR cells and its parental MCF-7 cells are sensitive to XN. Moreover, XN treatment decreased anti-apoptotic protein Bcl-2, pro-caspase 3, increased pro-apoptotic protein Bax, and induced apoptotic marker cleaved-PARP, and DNA damage marker γ-H2AX (Figure 2E,F), which was the same with the XN-induced apoptosis in MCF-7 cells [11], indicating XN also induced apoptosis in MCF-7/ADR cells. In addition, we also detected the effect of XN on the cell cycle of MCF-7/ADR cells, and we found XN could increase the percentage of cells in both S and G2/M phase and decrease the distribution in G0/G1 phase (Figure 2G), suggesting XN could also disturb the cell cycle distribution of MCF-7/ADR cells.

### 2.2. Synergic Effect of XN in Combination with DOX

**Figure 3:**
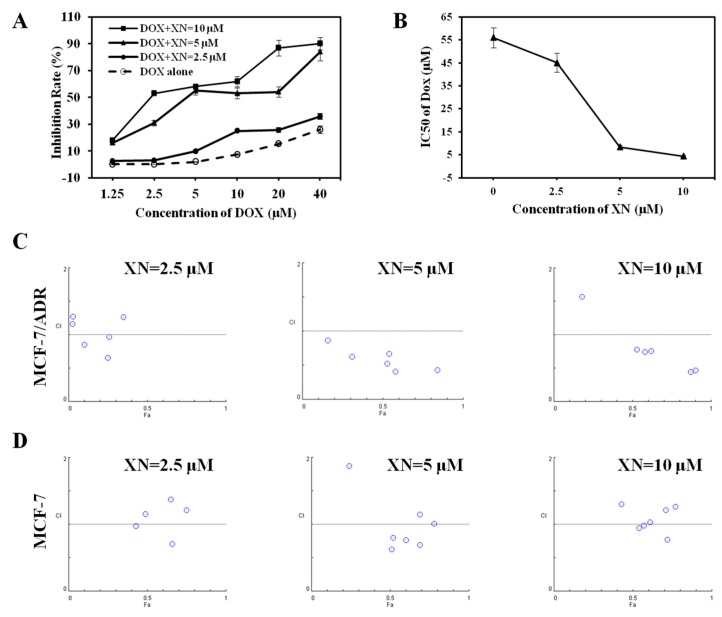
XN sensitizes the cytotoxicity of DOX in MCF-7/ADR cells. (**A**) MCF-7/ADR cells were treated with DOX in the absence and presence of increasing XN concentrations (2.5, 5, and 10 μM). After treatment of 72 h, the cells viability was analyzed by MTT assay; (**B**) XN decreases the IC_50_ values of DOX against MCF-7/ADR cells. Cells were treated with DOX for 72 h in the presence of 2.5, 5, and 10 μM XN and the IC_50_ values of DOX were calculated, respectively; (**C**) the Chou and Talalay CI analysis of synergistic effects with DOX. MCF-7/ADR cells were treated with DOX (0–40 μM) in the presence of XN (2.5, 5, and 10 μM) for 72 h; (**D**) The Chou and Talalay CI analysis of synergistic effects with DOX. MCF-7 cells were treated with DOX (0–10 μM) in the presence of XN (2.5, 5, and 10 μM) for 72 h.

In addition to used alone, XN could also enhance the sensitivity of MCF-7/ADR cells to DOX (Figure 3A). When treated with DOX alone, the IC_50_ value of DOX against MCF-7/ADR cells was 55.9 ± 4.3 μM. In the presence of XN 2.5, 5, and 10 μM, the IC_50_ values of DOX decreased to 45.1 ± 4.1, 8.5 ± 0.8, and 4.3 ± 0.3 μM, respectively (Figure 3B) and, thus, XN increased the sensitivity of MCF-7/ADR cells to DOX concentration-dependently. The synergic effect of DOX and XN on cell growth was further evaluated by isobolographic analysis method. As shown in Figure 3C, when DOX combined with XN at 2.5 μM, the CI values ranged from 0.66 to 1.27; when DOX combined with XN at 5 and 10 μM, most of the CI values ranged from 0.41 to 0.87, except one CI value, which was 1.56, suggesting the synergistic behavior between XN and DOX, especially at the relatively higher concentration of XN (5 and 10 μM). The CI values were listed in Table 1. When combined with DOX in the parental DOX sensitive MCF-7 cells, XN (2.5, 5, and 10 μM) also decreased DOX IC_50_ values from 1.44 ± 0.09 to 1.43 ± 0.12, 0.66 ± 0.04, 0.27 ± 0.01 μM, respectively, however, most of the CI values were larger than 1 (Table 2, Figure 3D), suggesting the synergistic behavior between XN and DOX in MCF-7 cells was not as obvious as in MCF-7/ADR cells.

### 2.3. XN Decreases the SP Cell Fraction in MCF-7/ADR Cells

**Figure 4:**
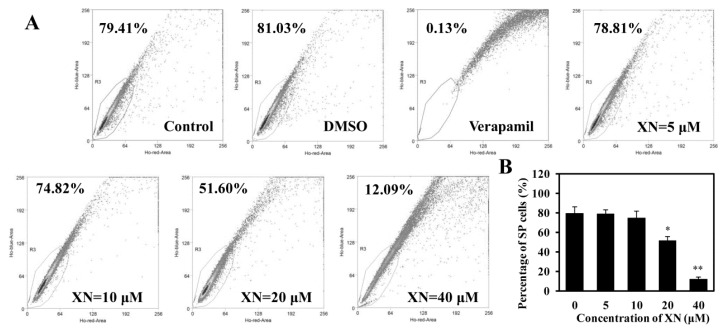
XN inhibits SP cell percentage in MCF-7/ADR. (**A**) A representative result of the SP cell percentage is displayed. After treatment with XN (0–40 μM) for 48 h, SP cells were counted by flow cytometry using Hoechst 33342 staining. Verapamil was used to set the SP gate; (**B**) histograms show the decreasing percentage of SP cells. Data shown are the means ± SD of three experiments for each group. * p < 0.05, ** p < 0.01 versus medium control.

SP cells are a sub-population cells that is distinct from the main population due to their resistance to the fluorescence dyes. Usually, SP cells contain a relatively high percentage of cancer stem cells, maintain the stemness, and contribute to the drug resistance of breast cancers [15]. To investigate whether XN could affect the SP cells and down-regulate the stemness, we examined the SP cells percentage after treated with XN. As shown in Figure 4A, pre-incubating with verapamil, the SP percentage decreased obviously, and the area where cells disappeared was used to set the SP gate. In the control group, the percentage of the SP cells was 79.41%. The percentage of the SP cells in MCF-7/ADR cells in the vehicle control DMSO group was 81.03%, however, the percentage decreased to 78.81%, 74.82%, 51.60%, and 12.09% after being treated with 5, 10, 20, 40 μM XN, respectively, in an obvious concentration-dependent manner (Figure 4B). Our data showed that XN significantly inhibited the SP cells percentage in MCF-7/ADR cells.

### 2.4. XN Reduces Clonogenic Survival, Migration, and Sphere Formation of CSC-Like Cells

**Figure 5:**
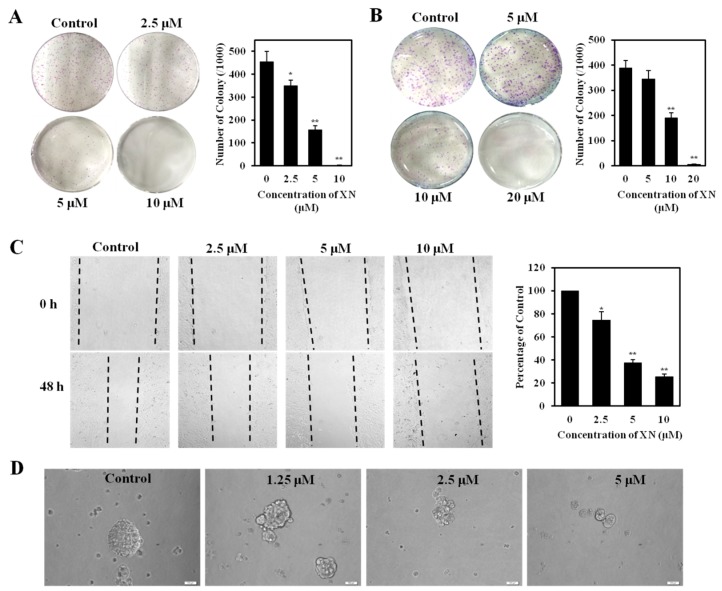
XN decreases the stemness of MCF-7/ADR. (**A**) XN inhibits the colony formation of MCF-7/ADR cells. Colony formation assay was performed as described in the Materials and Methods section. Shown is a representative from three experiments with similar results. The histogram shows the number of colonies in the presence and absence of XN. * p < 0.05, ** p < 0.01 versus medium control; (**B**) XN disturbs the preformed colonies of MCF-7/ADR cells. Values represent the means ± SD of triplicate measurements; * p < 0.05, ** p < 0.01 versus medium control; (**C**) XN inhibits the migration of MCF-7/ADR. Cells were treated with XN (0–10 μM) for 48 h, and analyzed with scratch-wound assay. Dotted lines show the migration edge of the cells. Histogram shows the relative migration distance in the presence and absence of XN. * p < 0.05, ** p < 0.01 versus medium control; (**D**) XN inhibits mammospheres formation in vitro. MCF-7 cells were cultured in ultra-low-attachment plates with serum-free medium and treated with XN for 25 days. Mammospheres were observed and photographed under microscope (×200, scale bar = 500 μm).

High clonogenicity is one of the characters of breast cancer stemness. To determine whether XN reduced the clonogenic survival of MCF-7/ADR cells, we performed the clonogenic assay. On the sixth day after treatment with XN, both the number and the diameter of MCF-7/ADR colony were significantly decreased compared to the control group (Figure 5A). Furthermore, we have observed that XN could also inhibit the growth of the preformed colonies. MCF-7/ADR cells were plated in the six-well plates for five days. The colonies formed and were incubated with XN for another six days. XN could inhibited the growth of the preformed colonies in a concentration-dependent manner, and a higher concentration of XN (20 μM) could destroy the preformed colonies completely (Figure 5B), further confirming XN reduced clonogenic survival of MCF-7/ADR cells. Higher motility is another character of CSC-like cells. We further examined the migration of MCF-7/ADR cells after XN treatment. As shown in Figure 5C, XN suppressed cell migration in a concentration-dependent manner and, therefore, inhibited the motility of MCF-7/ADR cells. Breast cancer stem-like cells have been demonstrated to be enriched in non-adherent spherical clusters of cells, termed mammospheres [16]. We next performed the mammosphere formation assay to further investigate the effect of XN on cancer stem-like cell population. After treated with XN (0–5 μM), the formation of mammospheres reduced concentration-dependently (Figure 5D) and, thus, inhibited the self-renewal ability of the cancer stem-like cells. All these results suggested XN could affect the stemness of MCF-7/ADR cells.

### 2.5. XN Reduces the Expression of Cancer Stem-Like Cell Markers

**Figure 6:**
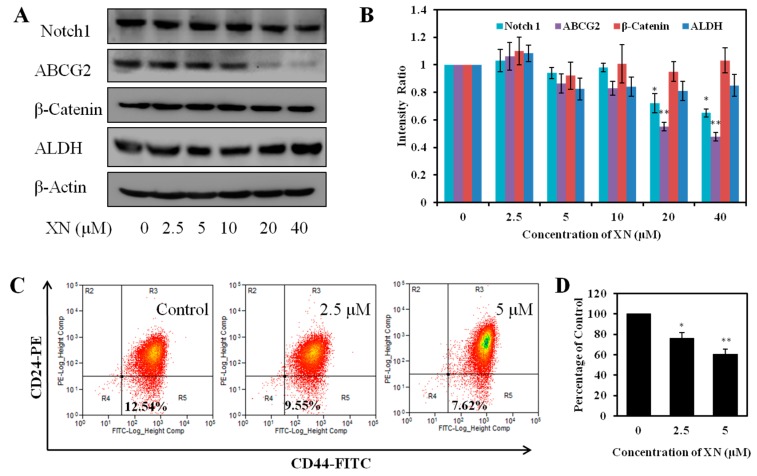
XN decreases the cancer stem-like cell markers of MCF-7/ADR. (**A**) XN regulates the expression of cancer stem-like cell markers. The biomarkers related to the stemness in MCF-7/ADR cells were measured by western blotting after treated with XN for 48 h; (**B**) protein levels quantified by scanning densitometry and normalized to control. Results are expressed as a ratio of control. Data represent means ± SD from three independent experiments. * *p* < 0.05, ** *p* < 0.01 for control cells versus XN treated cells; (**C**) XN reduces the percentage of CD44+/CD24− breast cancer stem-like cells. The phenotype of CD44+/CD24− cells was measured by flow cytometry after treatment with 5 and 10 μM XN for 48 h; (**D**) The histogram shows the decreased population of CD44+/CD24− cells. Results are normalized to untreated cells and the data shown are the means ± SD of three experiments for each group. * p < 0.05, ** p < 0.01 versus medium control.

To further confirm the effect on the stemness of MCF-7/ADR cells, stem cell markers were detected. We found that XN could decrease Notch 1 expression in MCF-7/ADR cells. Moreover, XN could markedly decrease the expression of ABCG2, while no obvious effect on the level of β-catenin and ALDH (Figure 6A,B). More specifically, the CD44+/CD24− cell population was analyzed to identify the effect of XN on MCF-7/ADR stem-like cells. After XN treatment for 48 h, cells were stained by CD44-FITC and CD22-PE, and phenotype of CD44+/CD24− cells was analyzed by flow cytometry. As shown in Figure 6C,D, the CD44+/CD24− cell percentage in the untreated MCF-7/ADR cells is 12.54%, while the percentage decreased to 9.55 and 7.62% after treated with 2.5 and 5 μM XN, respectively. These results indicated XN could regulate the expression of cancer stem-like cell markers and confirm the effect on MCF-7/ADR cells stemness.

## 3. Discussion

XN is a prenylated chalcone existing uniquely in hops, and is considered as a potential drug candidate for preventing and treating many kinds of cancers [9,10]. Because other groups have already reported XN could inhibit the viability and induce apoptosis in MCF-7 cells [10,11], here, we mainly focused to MCF-7/ADR cell line, which is resistant to many kinds of anticancer drugs, to evaluate the sensitivity of this cell line to XN. We revealed that XN could inhibit viability and induce apoptosis and thereof MCF-7/ADR was sensitive to XN. When detecting the apoptosis related proteins, including Bax, Bcl-2, pro-caspase 3, and cleaved-PARP, we observed the increased level of γ-H2AX (Figure 2C,D). In addition to the induction of apoptosis, this increased level of γ-H2AX also indicated the cytotoxic effect of XN possibly had relationship with its genotoxic effect. However, others reported that XN exhibits anti-genotoxic effects against many mutagens [8]. Therefore, the increased level of γ-H2AX is possibly the result of XN-induced apoptosis. Moreover, we also showed that XN possessed a potent synergism with DOX in MCF-7/ADR cells, and this finding was in line with the previous report that XN may also be a potent chemo- and radio-therapy sensitizer via STAT3 and EGFR suppression [11], however, in addtion to the synergism with chemotherapy, we also revealed that XN could down-regulate the cancer stemness characters in MCF-7/ADR cells, for the first time.

Breast cancer stem-like cells are characterized by their high clonogenicity, increased migration, and resistance to the fluorescence dyes. Conventional chemotherapeutic agents usually lead to an enrichment of SP cells, revealing their defect to target the SP cells. Thus, agents that can reduce the SP phenotype are currently in vogue in cancer therapeutics. The cytotoxicity in MCF-7/ADR and the synergism effect with DOX made us wonder whether XN could regulate the SP cells. As expected, XN markedly decreased the SP percentage in MCF-7/ADR cells. ABCG2 is traditionally considered as a membrane transporter that is responsible for SP cells by transporting Hoechst dye out of the stem cell membrane [17]. ABCG2 are more than just drug efflux pumps, but also a stem cell marker and regulates stem cell signaling at the upstream [18]. Whether XN could reduce the SP cells percentage via modulation on ABCG2 and other ATP binding cassette transporters needs further investigations. In addition to its effect on SP cells, we also observed that XN also down-regulated other stem-like characteristics of MCF-7/ADR, including the suppression of clonogenic and metastatic capacity, and the renewal ability. The metastatic capacity inhibition possibly has a relationship with XN down-regulataion in matrix metalloproteinases (MMPs) [19]. Notch pathway is inhibited by XN in hepatocellular carcinoma [20] and pancreatic cancer [21]. Considering Notch pathway is one of the major signaling pathways regulating cancer stem cells, we also detected Notch expression level in MCF-7/ADR cells, the results showed that, similar to other cell lines [20,21], XN could down-regulate the expression level of Notch. However, the detailed signaling pathway(s) and molecular mechanisms underlying the XN-mediated stemness regulation remain further more investigations. 

## 4. Materials and Methods

### 4.1. Materials and Cells

XN (purity > 98%) was purchased from Nanjing Spring and Autumn Biological Engineering Co., Ltd., Nanjing, China. Antibodies against γ-H2AX, Bax, Bcl-2, Notch, ABCG2, ALDH, β-Catenin, cleaved poly (ADP-ribose) polymerase (PARP), pro-caspase 3, and actins were purchased from Cell Signaling Technology Inc., Danvers, MA, USA,. Other reagents and kits were the products of Beyond, China. Human breast cancer MCF-7 were provided by the Cell Bank of Chinese Academy of Sciences, DOX resistant sub-line MCF-7/ADR was established by a stepwise increase of DOX concentrations in the culture form the parental human breast cancer cell line MCF-7 and maintained in our lab. Cells were cultured in RPMI-1640 medium (GIBCO, Grand Island, NY, USA) supplemented with 10% fetal bovine serum (FBS), penicillin, and streptomycin at 37 °C in 5% CO_2_. 

### 4.2. Cell Viability Assessment

The cytotoxicity of XN was determined by 3-(4,5)-dimethylthiahiazo(-z-y1)-3,5-di-phenytetrazoliumromide (MTT) assay. Briefly, cancer cells were untreated or treated with certain concentrations of XN for different periods. MTT was added and incubated for another 4 h, and then the dye crystals were dissolved in DMSO. Absorbance was measured at 490 nm.

### 4.3. Drug Combination and Calculation of Synergism

MCF-7/ADR cells were treated with DOX alone, XN alone, or with the combination of these two drugs, at the indicated doses. MTT assays were performed after 72 h of treatment. The medium-effect method was used to analyze concentration-response data for single drug or multiple drugs. The synergistic effect of multiple drugs was calculated by the definition of Chou and Talalay [22]. The Chou and Talalay combination index (CI), reflecting the interaction of two drugs, was calculated by Calcusyn (Biosoft, Cambridge, UK). The CI values of <1, 1, and >1 indicate synergistic, additive, and antagonistic effects, respectively.

### 4.4. Colony Formation Assay

MCF-7/ADR cells were plated onto six-well plates, at a density of 1000 cells per well. XN was added to medium 24 h or 5 days after seeding. After incubated with XN for additional six days, cells were washed, fixed, and stained with Giemsa. Colonies consisting of >50 cells were scored.

### 4.5. Cell Migration Assay

MCF-7/ADR cells were counted and plated in 24-well plates. After growth to 90% confluence, “scratch” wounds were created. After washing with phosphate buffer saline (PBS) twice, RPMI-1640 medium supplemented with 2% FBS containing certain concentrations of XN was added. After incubation for 48 h, three fields of each wound were selected and photographed. The relative width of the remaining wound was measured and the migration inhibition rate was calculated.

### 4.6. Side Population (SP) Cells Analysis

MCF-7/ADR cells were plated onto six-well plates and treated with XN (0–40 μM) for 48 h. Then, cells were collected and suspended in RPMI-1640 with or without verapamil (50 μM) for 10 min. Verapamil was used as a positive control to set the SP gate. Hoechst 33342 was then added to a final concentration of 2.5 μg/mL and incubated for 90 min at 37 °C. Cells were washed and finally suspended in ice cold PBS containing 2% FBS. PI (2 μg/mL) was added 5 min before the analysis to expel the dead cells in the sample. The percentage of SP cells was analyzed by flow cytometer moflo XDP (Beckman-Coulter, Miami, FL, USA).

### 4.7. Western Blotting Assay

After treated with XN, MCF-7/ADR cells were collected, washed, and lysed with radio-immunoprecipitation assay (RIPA) buffer containing fresh protease inhibitor mixture. Protein samples were resolved on 12% SDS-polyacrilamide gels, transferred to nitrocellulose membranes and probed with protein-specific antibodies, followed by HRP-conjugated secondary antibody. Bands corresponding to the antibodies were detected by FluorChem E (ProteinSimple, Santa Clara, CA, USA). Protein levels were quantified by scanning densitometry and normalized to control.

### 4.8. Mammosphere Formation Assay

The MCF-7 cells were plated in ultra-low-attachment 6-well plates at a density of 2000 cells/well in primary culture, which were supplemented with 2 mM l-glutamine, 2% B27 supplement, 10 ng/mL human recombinant epidermal growth factor and 20 ng/mL basic fibroblast growth factor, and 5 μg/mL insulin, and XN (0–5 μM). Mammospheres were observed under a microscope and photographs were acquired after culture for 21 days.

### 4.9. CD44 and CD24 Staining

The MCF-7/ADR cells were plated in six-well plates. After treatment of XN for 48 h, the MCF-7/ADR cells were stained with phycoerythrin-conjugated anti-human CD24 antibody and FITC-conjugated anti-human CD44 antibody according to the manufacturer’s instructions. Samples were analyzed using a flow cytometer moflo XDP (Beckman-Coulter).

### 4.10. Mammosphere Formation Assay

Cells were treated with certain concentrations of XN (0–20 µM) for 24 h. The cells were harvested and fixed in ice-cold 70% (*v*/*v*) ethanol for 24 h at 4 °C. The cell pellet was collected by centrifugation at 5000× *g*, resuspended in PBS, and stained with a mixture of RNase (10 μg/mL) and PI (50 μg/mL) in sodium citrate containing 0.5% Triton X-100 for 20 min in the dark. Cell cycle analysis was performed using flow cytometry and cell cycle distribution was analyzed with ModFit LT software (Verity Software House Inc., Topsham, ME, USA).

### 4.11. Data Analysis

One-way ANOVA with Tukey’s post hoc test was used for the statistical analysis of the data. The results were expressed as mean values ± SD. Differences of *p* < 0.05 were considered statistically significant.

## 5. Conclusions

In conclusion, we have confirmed that the DOX-resistant cell line MCF-7/ADR was sensitive to XN. Further insights were gained into the combination with DOX and XN, and we demonstrated that XN had the potent synergy with DOX. The mechanisms of the synergy effect were related to the down-regulation of the breast cancer stem-like cells characters, which has been reported for the first time. This study demonstrated that XN was an agent that could target the breast cancer stem-like cells. Therefore, it could be used alone or in combination with other anticancer agents for the treatment of DOX resistant breast cancer.

## Figures and Tables

**Table 1:** Result of isobolographic analysis showing CI of DOX in the presence of different concentrations of XN in MCF-7/ADR cells. The CI values were calculated by CompuSyn (ComboSyn, Inc., Paramus, NJ, USA).

XN (μM)	DOX (μM)	Effect	CI
2.5	1.25	0.026	1.16
2.5	2.5	0.03	1.27
2.5	5	0.1	0.85
2.5	10	0.25	0.66
2.5	20	0.26	0.97
2.5	40	0.35	1.26
5	1.25	0.16	0.87
5	2.5	0.31	0.62
5	5	0.58	0.41
5	10	0.53	0.52
5	20	0.54	0.67
5	40	0.84	0.42
10	1.25	0.18	1.56
10	2.5	0.53	0.77
10	5	0.58	0.74
10	10	0.62	0.75
10	20	0.87	0.44
10	40	0.90	0.47

**Table 2:** Result of isobolographic analysis showing CI of DOX in the presence of different concentrations of XN in MCF-7 cells. The CI values were calculated by CompuSyn.

XN (μM)	DOX (μM)	Effect	CI
2.5	0.15	0.19	1.89
2.5	0.3	0.22	2.33
2.5	0.625	0.43	0.97
2.5	1.25	0.49	1.16
2.5	2.5	0.66	0.71
2.5	5	0.65	1.38
2.5	10	0.75	1.21
5	0.15	0.24	1.87
5	0.3	0.51	0.63
5	0.625	0.52	0.80
5	1.25	0.6	0.76
5	2.5	0.69	0.69
5	5	0.69	1.15
5	10	0.78	1.01
10	0.15	0.43	1.30
10	0.3	0.54	0.94
10	0.625	0.57	0.98
10	1.25	0.61	1.03
10	2.5	0.72	0.77
10	5	0.71	1.21
10	10	0.77	1.26
